# RfaH contributes to maximal colonization and full virulence of hypervirulent *Klebsiella pneumoniae*

**DOI:** 10.3389/fcimb.2024.1454373

**Published:** 2024-09-17

**Authors:** Yichuan Qiu, Li Xiang, Ming Yin, Chengju Fang, Xiaoyi Dai, Luhua Zhang, Ying Li

**Affiliations:** ^1^ Department of Clinical Laboratory, Hospital of Chengdu Office of People’s Government of Tibetan Autonomous Region, Chengdu, Sichuan, China; ^2^ The School of Basic Medical Sciences, Southwest Medical University, Luzhou, Sichuan, China

**Keywords:** hypervirulent *Klebsiella pneumoniae*, rfaH, capsule, anti-phagocytosis, virulence

## Abstract

Hypervirulent *K. pneumoniae* (hvKp) have emerged as clinically important pathogens, posing a serious threat to human health. RfaH, a transcriptional elongation factor, has been regarded as implicated in facilitating the transcription of long virulence operons in certain bacterial species. In *K. pneumoniae*, RfaH plays a vital role in promoting CPS synthesis and hypermucoviscosity, as well as mediating bacterial fitness during lung infection. In this study, we aim to conduct a systematic investigation of the roles of rfaH in the survival, dissemination, and colonization of hvKp through *in vitro* and *in vivo* assays. We found that bacterial cells and colonies displayed capsule -deficient phenotypes subsequent to the deletion of *rfaH* in *K. pneumoniae* NTUH-K2044. We confirmed that *rfaH* is required for the synthesis of capsule and lipopolysaccharide (LPS) by positively regulating the expression of CPS and LPS gene clusters. We found that the Δ*rfaH* mutant led to a significantly decreased mortality of *K. pneumoniae* in a mouse intraperitoneal infection model. We further demonstrated that the absence of *rfaH* was associated with slower bacterial growth under conditions of low nutrition or iron limitation. Δ*rfaH* displayed reduced survival rates in the presence of human serum. Besides, the engulfment of the Δ*rfaH* mutant was significantly higher than that of NTUH-K2044 by macrophages *in vivo*, indicating an indispensable role of RfaH in the phagocytosis resistance of hvKp in mice. Both mouse intranasal and intraperitoneal infection models revealed a higher bacterial clearance rate of Δ*rfaH* in lungs, livers, and spleens of mice compared to its wild type, suggesting an important role of RfaH in the bacterial survival, dissemination, and colonization of hvKp *in vivo*. Histopathological results supported that RfaH contributes to the pathogenicity of hvKp in mice. In conclusion, our study demonstrates crucial roles of RfaH in the survival, colonization and full virulence of hvKp, which provides several implications for the development of RfaH as an antibacterial target.

## 1 Introduction

*Klebsiella pneumoniae* is a Gram-negative strain that frequently causes pulmonary infection, urinary tract infections, bacteremia, and liver abscesses (Paczosa and Mecsas, 2016). Hypervirulent *K. pneumoniae* (hvKP) is associated with severe infections and exhibits an extremely high invasive and transmissible ability, causing higher morbidity and mortality for infected patients (Thomas A. Russo, 2019). hvKp strains may evolve into highly drug-resistant hvKp by acquiring various drug-resistant plasmids (Tian et al., 2022). Especially in recent years, the emergence of multi-resistant or even pan-resistant *K. pneumoniae* has significantly increased the risk of conversion of hvKp to highly drug-resistant hvKp, such as carbapenems-resistant hvKp isolates (Zhang et al., 2020; Yang et al., 2022), which poses a formidable challenge to human health globally.

RfaH is an antiterminator that directly binds to RNA polymerase (RNAP) and modifies it into a processive, pause-resistant state, thus reducing transcriptional pausing at certain positions and increasing RNAP processivity (Svetlov et al., 2007; Burmann et al., 2012). It specifically promotes the transcription of long pathogenicity operons that encode extracytoplasmic components in bacteria, such as adhesins, capsular polysaccharides, and toxins (Artsimovitch and Landick, 2002; Nagy et al., 2002). RfaH was initially revealed as a regulator of lipopolysaccharide (LPS) synthesis in *Salmonella* (Lindberg and Hellerqvist, 1980) and *Escherichia coli* (Creeger et al., 1984). It was also identified to regulate the F plasmid (Sanderson and Stocker, 1981), various types of the capsule (Clarke et al., 1999), hemin receptor (Nagy et al., 2002), and the α-hemolysin in *E.coli* (Stevens et al., 1994).

Numerous genetic factors contribute to the pathogenicity and virulence of *K. pneumoniae* strains, such as variable capsular polysaccharide (CPS) and LPS, siderophore, enterobactin, and fimbriae, are basic requirements for establishing opportunistic infections (Wyres et al., 2020). Although a hypermucoviscosity (HMV) colony phenotype is not pathognomonic for hvKp, it has been strongly associated with hvKp strains (Walker and Miller, 2020). Previous studies hinted that RfaH is required for the CPS synthesis and HMV (Mike et al., 2021), and is critical for *K. pneumoniae* fitness in the lung, by functioning in resisting complement-mediated serum killing, and for maximal growth in serum (Bachman et al., 2015; Short et al., 2020). However, a research gap exists regarding the regulatory roles of RfaH in the pathogenicity and virulence of *K. pneumoniae*, especially in hvKp.

In this study, we showed that the deletion of *rfaH* caused significant attenuation of virulence in *K. pneumoniae* NTUH-K2044, a reference strain of serotype K1 that is highly virulent and hypermucoviscous (Wu et al., 2009). We demonstrate that RfaH contributes to pathogenicity by facilitating bacterial growth in low nutrition and iron-limited conditions, and by regulating bacterial evasion of complement-mediated killing and phagocytosis by macrophages. Especially, we further clarified that RfaH plays critical roles in helping bacterial dissemination, colonization, and full virulence of *K. pneumoniae* by two different mouse infection models.

## 2 Materials and methods

### 2.1 Bacterial strains, plasmids, media, and growth condition

Bacterial strains and plasmids used in this study are listed in **Table 1**. The *K. pneumoniae* NTUH-K2044, a strain that was isolated from the blood of a patient with community-acquired primary liver abscess and metastatic meningitis, was kindly gifted by professor Jin Town Wang of the National Taiwan University Hospital, Taipei, Taiwan. All the test strains were cultured in Luria-Bertani (LB) broth with 220 rpm shaking or on LB agar plates at 37°C.

**Table 1:** Bacterial strains and plasmids used in this study.

Strains or plasmids	Descriptions	References or source
*E. coli*		
DH5α	Cloning host	Laboratory stock
*K. pneumoniae*		
NTUH-K2044	Clinical isolate of K1 serotype	(Wu et al., 2009)
Δ*rfaH*	NTUH-K2044 with deletion of *rfaH*	This study
Δ*rfaH*-comp	Δ*rfaH* with complementation of *rfaH*	This study
Plasmids		
pKO3-km	Km^r^, suicide vector	(Pan et al., 2008)
pKO3-km-*rfaH* L/R	Km^r^, suicide vector for *rfaH* deletion	This study
pKO3-km-*pgpA*-*yajO*-*rfaH*	Km^r^, suicide vector for *rfaH* complementation	This study

### 2.2 Construction of the Δ*rfaH* mutant and its complementation Δ*rfaH*-comp

The primers for construction of mutant and complementation strains are listed in **Supplementary Table S1**. To delete *rfaH*, a ~1kb DNA fragment of the upstream and downstream regions flanking *rfaH* were PCR amplified from the chromosome of NTUH-K2044, and ligated into the suicide vector pKO3-Km that was cut with *Not*I (New England Biolabs) using the ClonExpress II One Step Cloning Kit (Vazyme, China). The resulting recombinant plasmid pKO3-km-*rfaH* L/R was transformed into NTUH-K2044 by electroporation and allowed to recover for 1 h at 30°C. The chromosomal integrates were selected on LB plates containing 50 μg/ml kanamycin after incubation at 43°C for ~16h. After the first round of DNA allelic exchange, the whole recombinant plasmid was expected to have incorporated into the chromosome. To select cells in which the *rfaH* gene was excised and the plasmid was lost, colonies from the first round DNA exchange event were diluted and simultaneously plated on LB plates containing 10% sucrose and those containing 50 μg/ml kanamycin, respectively. After incubation at 30°C for ~24h, kanamycin-sensitive colonies were picked and the deletion of *rfaH* was confirmed by PCR and DNA Sanger sequencing using gene specific and genome flanking primers. Comparative analysis of sequencing results was performed by BLASTn. The resulting mutant was denoted as Δ*rfaH*. For complementation, the intact *rfaH* gene containing its native promoter was amplified by PCR and cloned into the intergenic region between open reading frames (ORFs), *pgpA* and *yajO*, using a pKO3-Km *pgpA*-*yajO* recombinant vector (Hsieh et al., 2010). The recombinant plasmid pKO3-km-*pgpA*-*yajO*-*rfaH* was transformed into Δ*rfaH* by electroporation. After two rounds of homologous recombination, the chromosomal integration of *rfaH* was selected by LB plates containing 50 μg/ml kanamycin and those containing 10% sucrose, respectively. After PCR analysis and Sanger sequencing, the resulting complemented strain was named Δ*rfaH*-comp.

### 2.3 *In vitro* growth assays

NTUH-K2044, Δ*rfaH* and Δ*rfaH*-comp were cultured overnight at 37°C. For LB growth experiment, a fresh culture was inoculated at 1:100 from the overnight culture and grown at 37°C for 36 h in 96-well plates. For M9 minimal medium growth experiment, overnight culture of the tested strains were sub-cultured at 1:100 to M9 broth and grown at 37°C for 18 h in 96-well plates. For the iron limitation growth experiment, the tested strains were sub-cultured at 1:100 in LB broth containing 100, 200, 400, or 800 μM 2,2’-dipyridine (Sangon, Shanghai, China), respectively, and grown at 37°C for 12 h in 96-well plates. The bacterial growth status was automatically monitored by Synergy H1 Hybrid Multi-Mode Reader (BioTek, America) at optical density 600 nm (OD600 nm).

### 2.4 *In vitro* stress assays

*In vitro* stress assays were performed as described previously with slight modifications (Hsieh et al., 2010; Srinivasan et al., 2012; Bulger et al., 2017). NTUH-K2044, Δ*rfaH* and Δ*rfaH*-comp were grown to mid-exponential phase (OD600 nm reaches 0.6-0.8). Cells were pelleted by centrifuging at 10,000 × g for 5 min and resuspended in LB broth. For the oxidative stress assays, cells were treated with or without 0.5 mM H_2_O_2_ for 30 min. For the temperature stress assays, cells were incubated at 40°C, 50°C, and 60°C for 20 min, respectively. For the acid stress assays, cells were resuspended in LB broth at pH 5.5 or 7.4 for 1 h, followed by centrifugation and resuspension in LB broth at pH 3.0 for 1 h. Cultures of these stress assays were diluted serially and spread onto LB plates for colony counting. For the osmotic stress assays, pellet cells were diluted serially and spread on LB plates with or without 0.3 M potassium chloride. The results are expressed as the ratio of the number of colony-forming units (CFUs) obtained after stress treatment to the number of CFUs obtained from untreated cultures. All these experiments were independently performed three times.

### 2.5 String test and sedimentation assay

The string test was carried out by stretching a mucoviscous string from the colony grown on a blood agar plate using a sterile loop as previously described (Fang et al., 2004). The viscous string >5 mm was identified as a positive result. The sedimentation assay was performed to further measure the levels of HMV as described previously (Palacios et al., 2018). Briefly, the tested strains cultured in LB broth (OD600 nm ~1.0) were centrifuged for 5 min at 1,000 × g, respectively. The supernatant was gently removed without disturbing the pellet for OD600 measurement.

### 2.6 Transmission electron microscopy

NTUH-K2044 and Δ*rfaH* were cultured overnight, and then sub-cultured at 1:100 to 15 ml fresh LB broth for 6 h, centrifuged at 1,0000 × g for 5 min, and filled with 3% glutaraldehyde fixative. The samples were observed under the transmission electron microscope JEM-1400FLASH (Japan Electronics, Japan).

### 2.7 Quantification of CPS and LPS

Capsule was extracted as previously described (Rosen et al., 2016; Walker et al., 2019). NTUH-K2044, Δ*rfaH*, and Δ*rfaH*-comp were cultured overnight, and uronic acid (UA) was extracted from 500 μl culture with Zwittergent 3-14 in 100 mM citric acid. The culture was then precipitated with absolute ethanol and resuspended in tetraborate/sulfuric acid. Following the addition of 3-hydroxy diphenol in 0.5% NaOH, the amount of uronic acid was determined by measuring absorbance at 520 nm. A standard curve was generated with glucuronic acid.

LPS was extracted by the hot-phenol water method with minor modifications (Lee and Inzana, 2021). Briefly, NTUH-K2044, Δ*rfaH* and Δ*rfaH*-comp were cultured overnight, followed by washing with PBS and resuspension in ddH_2_O. Following ultrasonication, an equal volume of pre-warmed 90% (w/v) phenol solution was added to the bacterial suspension. Stir the mixture vigorously at 68°C for 30 min, and collect the supernatant by centrifugation at 10,000 × g for 10 min. To precipitate the LPS, one-tenth volume of 5 M NaCl, and 5-10 volumes of cold (-20°C) 95% ethanol were added, and the mixture was kept at -20°C overnight. After centrifugation at 2,000 × g for 10 min, suspending the opaque pellet in 1mL of distilled water, followed by digestion with proteinase K at 59°C for 3h. The resulting LPS sample was stored at -20°C. The phenol sulfate method was used for LPS quantification. 1 ml of LPS sample was added with 1 ml ddH_2_O and 6% phenol, and 5 ml concentrated sulfuric acid. The amount of LPS was detected by measuring absorbance at 490 nm and determined from the standard curve. The LPS was applied to 12% sodium dodecyl sulfate-polyacrylamide gel electrophoresis (SDS-PAGE) and were stained using the silver staining method.

### 2.8 Biofilm formation

The biofilm formation assays were performed as previously described (Li et al., 2016). Briefly, overnight culture of the test strains was diluted at 1:100 into fresh LB and inoculated statically in sterile 96-well microtiter plates at 37°C for 12 h. The cultures were removed and the wells were washed by immersing them in water, followed by fixing with methanol and staining with crystal violet (0.1%, w/v) per well. After that, crystal violet was removed from wells and washed with ddH_2_O. The bound dye was released by adding 100 µl of acetic acid (33%, v/v) and the optical density of dissolved material was determined by measuring absorbance at 595 nm.

### 2.9 Serum bactericidal assay

The serum bactericidal assay was performed as described previously with minor modifications (Podschun et al., 1993). Briefly, NTUH-K2044, Δ*rfaH* and Δ*rfaH*-comp were grown to mid-exponential phase. We mixed 25 μL of each bacterial strain with 75 μL fresh or heat-inactivated (56°C for 30 min) human serum with informed consent from healthy volunteers. The serum-strain mixture was incubated at 37°C for 1h without shaking, and CFUs were enumerated by serial dilution and plating on LB agar plates. The survival percentage in serum was calculated by the ratio of the number of colonies in the fresh serum treatment group to the number of colonies in the heat-inactivated serum treatment group.

### 2.10 Quantitative reverse-transcription PCR

2 ml of NTUH-K2044 and Δ*rfaH* were grown to an OD600 of 0.6-0.8. Total RNA extraction and reverse transcription of RNA were carried out using the MiniBEST Universal RNA Extraction Kit (TaKaRa, Japan, and the PrimeScript™ RT reagent Kit with gDNA Eraser (TaKaRa, Japan), respectively, according to the manufacturer’s instructions. The primers for qRT-PCR are listed in **Supplementary Table S2**. qRT-PCR amplification was performed as follows: pre-denaturation (95°C for 30 s), cyclic reactions (40 cycles of 95°C for 10 s, 60°C for 30 s), and melting curve (95°C for 15 s, 60°C for 1 min, 95°C for 15 s). qRT-PCR reactions were conducted using the LineGene 9600 Plus PCR Detection System (BIOER, China). All experiments were repeated for three times, independently, and were performed in triplicate. *K. pneumoniae 16s rRNA* was used as a reference gene for normalization. Data were analyzed using the 2^-ΔΔCt^ method (Schmittgen and Livak, 2008).

### 2.11 Mouse infections

Animal experiments were approved by the Southwest Medical University Institutional Animal Care and Use Committee (Project license: swmu20220089). Four- to six-week-old BALB/c mice (SPF Biotechnology Co., Ltd, Beijing, China) were used in this study. Before and following inoculation, mice had unlimited access to food and water.

To detect whether the virulence of Δ*rfaH* was attenuated, we used a mouse intraperitoneal infection model as previously described with minor modifications (Russo et al., 2021). Briefly, 10 mice were randomly selected and were intraperitoneally inoculated with 100 μl bacterial suspension (~ 1 × 10^5^ CFU) of NTUH-K2044 or Δ*rfaH*. Three mice injected with 100 μl sterile PBS alone were used as a negative control. The mental state and death of the mice were monitored at 12-h intervals for 7 days.

For the phagocytosis assays, mice (five per group) were lightly anesthetized with isoflurane and then inhaled with 50 μl of bacterial suspension (~ 1 × 10^8^ CFU) via the intranasal route. At 90 min after inoculation, the trachea of mice were dissected and intubated with the catheter (38mm length, 20 gauge), and lavaged with 1 ml PBS to collect alveolar phagocytes 3 times. Suspensions were treated with 50 μg/ml of gentamicin at 37°C for 1 h, followed by centrifugation at 4000 × g for 5 min and resuspension in 1 ml PBS. The phagocytes were lysed using 1% TritonX-100 solution, vortexed for 15 s, serially diluted, and plated for bacterial enumeration.

In the intranasal infection model, mice were intranasally infected with NTUH-K2044 or Δ*rfaH* as previously described with minor modifications (Sharma et al., 2017; Palacios et al., 2018). Briefly, mice (twelve per group) were lightly anesthetized with isoflurane, followed by inhalation with 50 μl of bacterial suspension (~ 1 × 10^5^ CFU). The control mice were inhaled with 50 μl of PBS. Inoculated mice were monitored daily for two days, and their activity, breath rate, posture, diarrhea, hair, eyes, nose, and weight were evaluated as health-scoring criteria. For the organ burden assays, four mice were euthanized with pentobarbital sodium at a dose of 150 mg/kg at the indicated time points (6 h, 24 h, and 48 h). After that, the lungs, livers, and spleens of mice in each group were removed, weighed under sterile conditions, and homogenized in 1 ml PBS, followed by serial dilution for the enumeration of CFU on LB agar. The number of CFU detected in the organs was standardized per 0.1 g of wet organ weight. For histopathology, mice (three per group) were inhaled with 50 μl of bacterial suspension (~ 1 × 10^5^ CFU), and the control mice were inhaled with 50 μl of PBS. Inoculated mice were monitored and euthanized by injection of pentobarbital sodium after 24 h post-infection. Lungs, livers, and spleens of mice were removed and fixed with 10 volumes of 10% neutral-buffered formalin, embedded in paraffin, sectioned, and stained with hematoxylin-eosin (HE).

For the organ burden assay in the intraperitoneal infection model, mice (twelve per group) were injected with 100 μl of bacterial suspension (~ 1 × 10^4^ CFU), with the control mice being injected with 100 μl of PBS. Inoculated mice were monitored daily for two days, and their activity, breath rate, posture, diarrhea, hair, eyes, nose and weight were evaluated as health-scoring criteria. Four mice were euthanized with pentobarbital sodium at the indicated time points (6 h, 24 h, and 48 h post-infection). For the organ burden assays, the lungs, livers, and spleens of mice in each group were removed, weighed, homogenized, serially diluted, and plated on LB agar for colony counts as described above. For histopathology, mice (three per group) were inhaled with 50 μl of bacterial suspension (~ 1 × 10^4^ CFU). Lungs, livers, and spleens of mice were removed at 24 h post-infection, embedded in paraffin, and stained with HE.

### 2.12 Statistical analysis

Data were presented as means ± standard deviation (SD) and were analyzed with GraphPad 9.0 software. Two tailed unpaired student’s *t*-test was employed to determine the difference between the two groups. A two-tailed Mann-Whitney U test was used for data analysis in the organ burden assays. A log-rank (Mantel-Cox) test was used for the analysis of the mouse survival curve. The statistical significance was set to *P < 0.05, **P < 0.01, ***P < 0.001, and ****P < 0.0001.

## 3 Results

### 3.1 Construction of *rfaH* deletion mutant and morphological analysis

**Figure 1:**
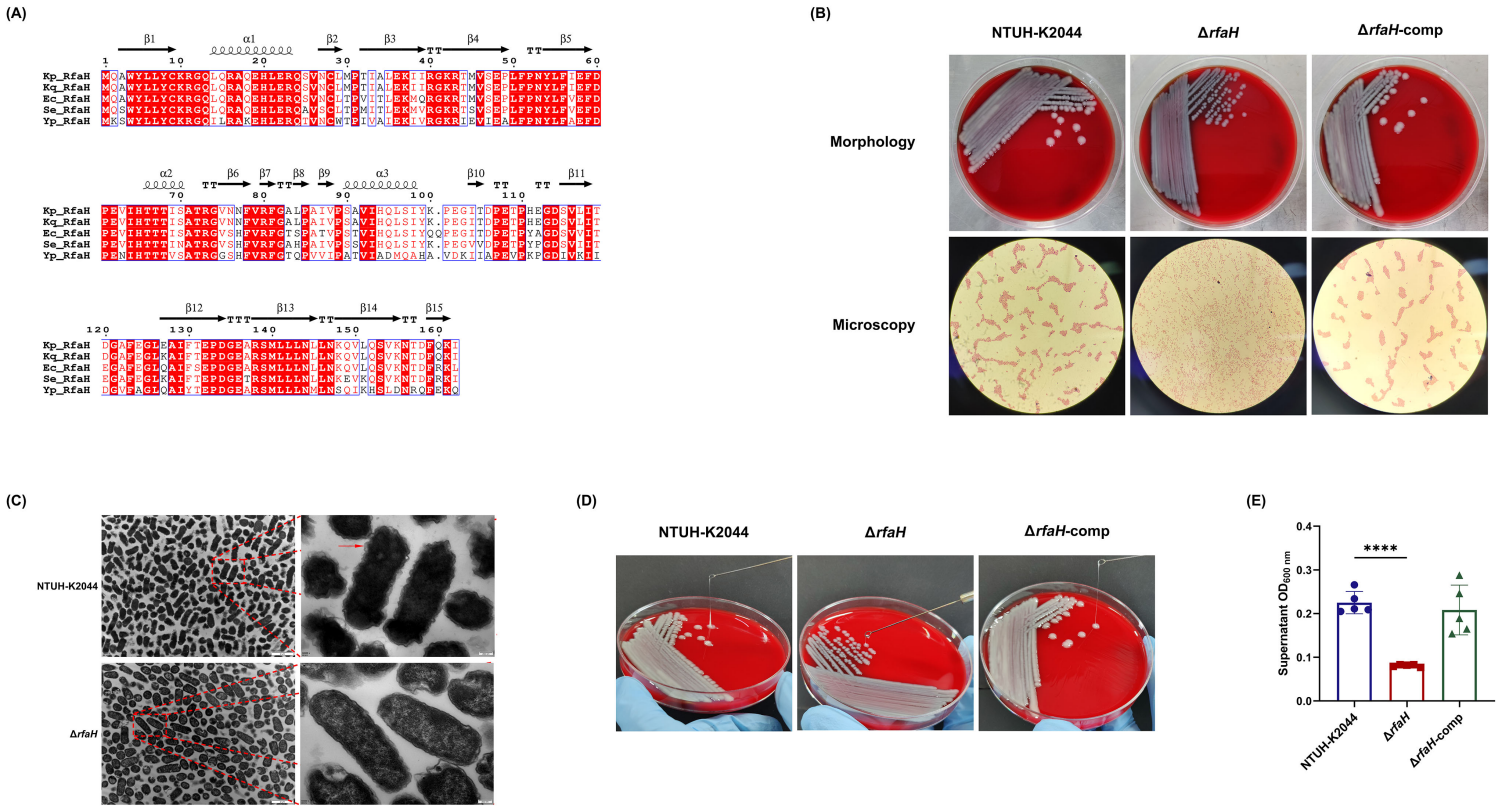
Construction and morphology characteristics of Δ*rfaH*. **(A)** Alignment of amino acid sequences of RfaH proteins from *Klebsiella quasipneumoniae* (Accession no. WP_131081528), *Escherichia coli* (MWO99696), *Salmonella enterica* (EEO3944103), and *Yersinia pestis* (WP_016594782). Analysis was performed using the ESPript 3.x (https://espript.ibcp.fr/ESPript/cgi-bin/ESPript.cgi). Secondary structures of Kp_RfaH predicted by SWISS-MODEL are indicated by coils for -helices and arrows for -strands. The background of identical residues is marked in red, and homologous residues are highlighted in red. **(B)** Representative colony phenotypes on the blood agar plates and the optical microscope examination (Gram-staining, 100×10) of NTUH-K2044, Δ*rfaH*, and Δ*rfaH*-comp. **(C)** Transmission electron microscope images of NTUH-K2044 and Δ*rfaH*. The red arrow indicates the capsule. **(D)** The string test. The viscous string of NTUH-K2044 and Δ*rfaH*-comp were positive, measuring >5 mm; the viscous string of Δ*rfaH* was negative, measuring <5 mm. **(E)** HMV assay by low-speed centrifugation. Data represent the means of at least three independent experiments, and error bars represent the standard error of the mean. Student’s *t*-test was performed for statistical analyses, ****P < 0.0001.

RfaH protein of *K. pneumoniae* NTUH-K2044 (162aa) encoded by the 489-bp *rfaH* gene, exhibited 99.38%, 86.5%, 83.95%, and 62.11% identity to RfaH in *Klebsiella quasipneumoniae* (Accession no. WP_131081528), *E. coli* (MWO99696), *Salmonella enterica* (EEO3944103), and *Yersinia pestis* (WP_016594782), respectively, by BLASTp analysis (**Figure 1A**). To determine the roles of RfaH, a *rfaH* deletion mutant was created by homologous recombination using the suicide plasmid pKO3-km in the wild-type *K. pneumoniae* NTUH-K2044. The genetic complementation of *rfaH* was done by integrating an intact *rfaH* gene containing its native promoter into the intergenic region between *pgpA* and *yajO* on the chromosome *of* Δ*rfaH.* PCR followed by DNA sequencing was performed to confirm the deletion and complementation of *rfaH.*

A distinct colony morphology was observed between NTUH-K2044 and Δ*rfaH*: the colony of NTUH-K2044 was large (3.2 ± 0.17 mm on average), raised, moist, and mucus, while Δ*rfaH* appeared as a non-mucus, smaller (1.83 ± 0.06 mm on average), colorless, and flat colonies (**Figure 1B**). Δ*rfaH*-comp had a similar colony morphology to that of the wild-type strain. Visualization of cells under optical microscope showed that NTUH-K2044 and Δ*rfaH*-comp cells formed distinct aggregates, while Δ*rfaH* cells displayed a dispersed arrangement (**Figure 1B**). Ultrastructural examination by TEM revealed that NTUH-K2044 was rough, with thick extracellular polysaccharides on the surface, whereas the Δ*rfaH* mutant was smooth, lacking of the capsule on the surface (**Figure 1C**). CPS production and HMV are closely related (Mike et al., 2021). The viscous string from the colonies of Δ*rfaH* was 1.33 ± 0.58 mm in length, indicating a negative string test result, while NTUH-K2044 generated 22.0 ± 4.0 mm string (**Figure 1D**). Sedimentation assay confirmed that Δ*rfaH* has reduced HMV, as the supernatant of NTUH-K2044 had an OD600 of approximately 0.225 ± 0.023 after centrifugation, while that of the Δ*rfaH* appeared clearer, measuring around 0.081 ± 0.0024 (Δ*rfaH* vs NTUH-K2044, *P*<0.0001) (**Figure 1E**). Complementation of *rfaH* restored HMV. These results suggest that RfaH is required for maintaining the hypercapsule morphology of bacterial cells and colonies, and is linked to the HMV phenotype.

### 3.2 RfaH contributes to the synthesis of CPS and LPS in hvKp

**Figure 2:**
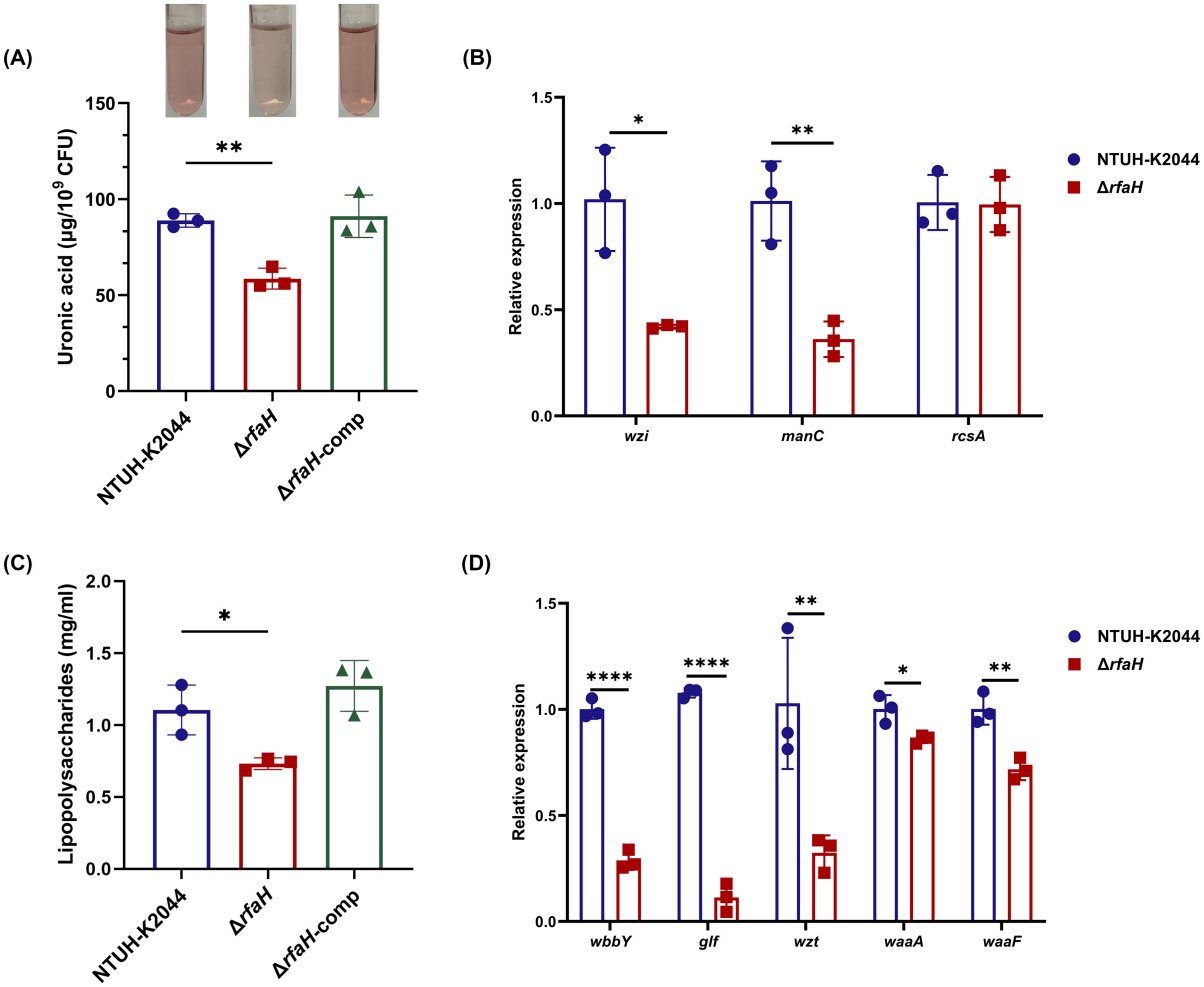
Δ*rfaH* produces less CPS and LPS. **(A)** Quantification of the capsule by the uronic acid assay. A representative result of this experiment is shown above the bar chart. **(B)** The transcription levels of genes associated with capsule production (*wzi*, *manC*, and *rcsA*) in NTUH-K2044 and Δ*rfaH*. **(C)** Quantification of LPS with phenol-sulfuric acid method; **(D)** The transcription levels of genes associated with LPS O-antigen (*wbbY*, *glf*, and *wzt*), and core lipopolysaccharide (*waaA*, and *waaF*) in NTUH-K2044 and Δ*rfaH*. Transcript abundance was measured using the 2^-ΔΔCt^ method with *16s rRNA* as a reference gene, and was normalized to the NTUH-K2044. Data represent the mean of at least three independent experiments (in triplicate), and error bars represent the standard error of the mean. *P* values were calculated using student’s *t*-tests for statistical analyses, *P < 0.05, **P < 0.01, ****P<0.0001.

The morphology changes of bacterial cells and colonies are most likely attributed to the impaired CPS biosynthesis in Δ*rfaH* (Mike et al., 2021). In this study, we confirmed that Δ*rfaH* displayed diminished UA content, measuring at 54.92 ± 0.95 μg/10^9^ CFU, while NTUH-K2044 (88.91 ± 2.81 μg/10^9^ CFU, Δ*rfaH* vs NTUH-K2044, *P*=0.0012) and Δ*rfaH*-comp (91.09 ± 9.02 μg/10^9^ CFU, Δ*rfaH* vs Δ*rfaH*-comp, *P*=0.0102) produced approximately 30-40% more UA (**Figure 2A**), confirming an essential role of *rfaH* in the CPS production of *K. pneumoniae.* We further assessed the transcription levels of capsule synthesis genes *wzi* and *manC* (Ares et al., 2016). Among them, the expressions of *wzi* and *manC* exhibited 58.7% (*P*=0.013) and 64.3% (*P*=0.0053) reduction, respectively, in Δ*rfaH* when compared to the wild-type strain (**Figure 2B**). These results suggest that RfaH promotes CPS production by positively regulating the expression of *cps* gene cluster containing *wzi* and *manC*. It has been shown that RcsA could activate *cps* gene expression in numerous strains (Su et al., 2018; Walker and Miller, 2020). While, we found no significant difference in the expression levels of *rcsA* between Δ*rfaH* and its parent strain (*P*=0.9313, **Figure 2B**).

It has been shown that RfaH enhances the transcription of a select group of operons controlling bacterial surface features, including LPS. Therefore, we quantified the LPS and found that its content in Δ*rfaH* (0.732 ± 0.034 mg/ml) was significantly lower than that of NTUH-K2044 (1.105 ± 0.141 mg/ml, *P*=0.0219) (**Figure 2C**). Complementation of *rfaH* restored the LPS to the wild-type level (1.273 ± 0.144 mg/ml, Δ*rfaH* vs Δ*rfaH*-comp, *P*=0.0067). Additionally, SDS-PAGE silver staining also revealed a lower level of LPS in Δ*rfaH* when compared to NTUH-K2044 and Δ*rfaH*-comp (**Supplementary Figure S1**). qRT-PCR showed that the transcription levels of O-antigen genes *wbbY*, *glf*, and *wzt* were reduced by 71.3% (*P*<0.0001), 89.5% (*P*<0.0001), and 68.5% (*P*=0.0029), respectively, in Δ*rfaH* compared to the wild-type strain. Meanwhile, the expression of core lipopolysaccharide genes *waaA* and *waaF* also showed 14.06% and 28.36% reduction (Δ*rfaH* vs NTUH-K2044, *P*=0.0239, and *P*=0.0054, respectively) (**Figure 2D**), confirming the positive regulation role of *rfaH* in the expression of O-antigen biosynthesis enzymes and core lipopolysaccharide. These results indicate that RfaH promotes the synthesis of LPS in *K. pneumonia*, as reported in *E. coli* and *Yersinia* sp (Singh et al., 2014; Hoffman et al., 2017).

### 3.3 The virulence of Δ*rfaH* is significantly attenuated

**Figure 3:**
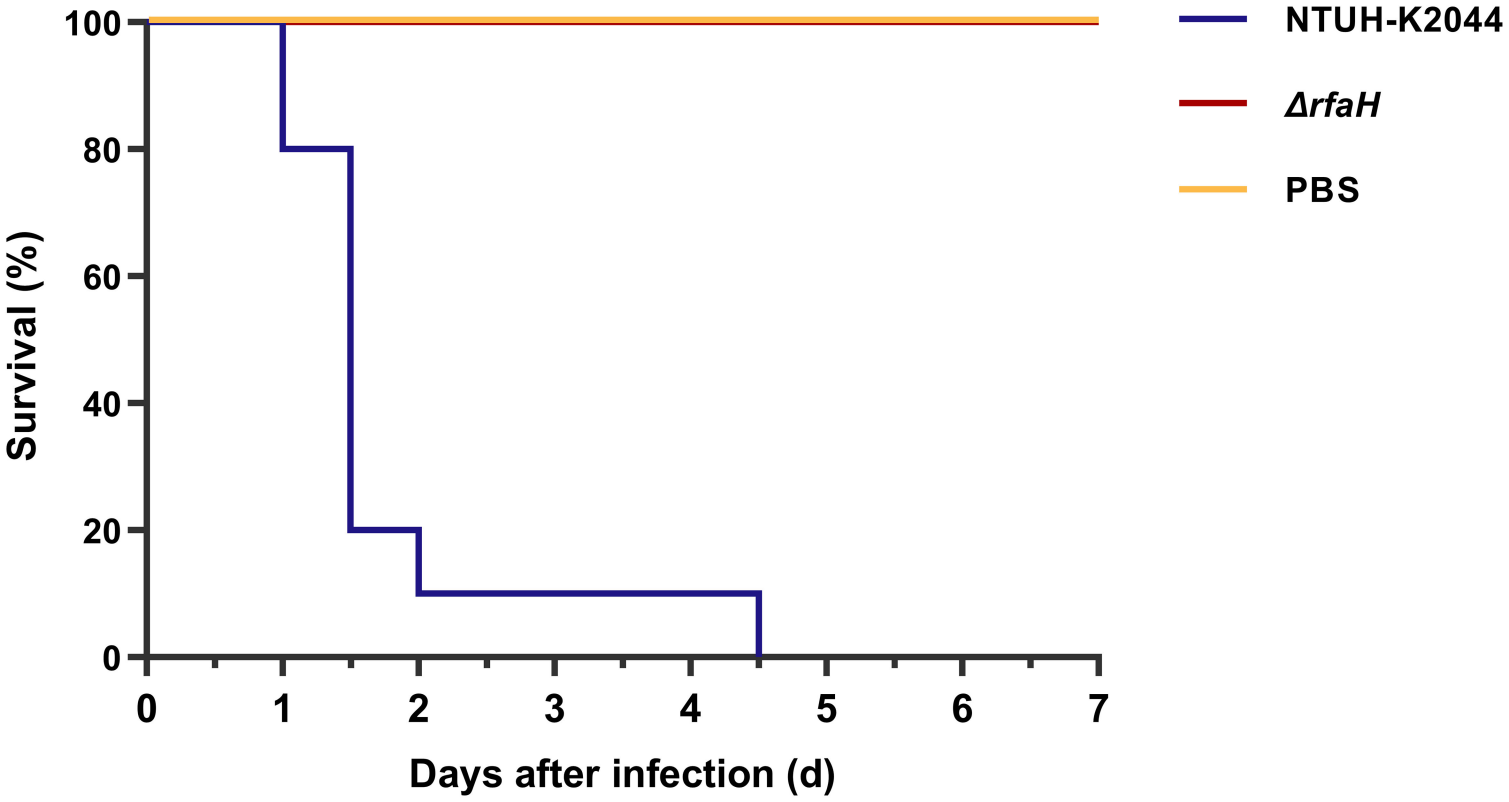
Δ*rfaH* is significantly attenuated in virulence. Survival curves of mice intraperitoneally inoculated with 1 × 10^5^ CFU of NTUH-K2044 or Δ*rfaH* show that the survival rate of Δ*rfaH-*infected mice was significantly enhanced (*P* < 0.0001, by a log-rank [Mantel-Cox] test).

To investigate whether the deletion of *rfaH* would affect the virulence of *K. pneumoniae* NTUH-K2044, we intraperitoneally inoculated BALB/c mice with ~1×10^5^ CFU of Δ*rfaH* or NTUH-K2044 and monitored survival. After being infected with NTUH-K2044, 20% mice died within 24 h, 90% died within 48 h, and 100% died at 4.5 days. While all mice survived the injection of Δ*rfaH* over 7 days (**Figure 3**). These data show a significant attenuation of virulence after the deletion of *rfaH* (*P* < 0.0001), supporting the role for RfaH in contributing to the virulence of *K. pneumoniae*.

### 3.4 RfaH is required for bacterial growth in low-nutrition and iron-limited conditions

**Figure 4:**
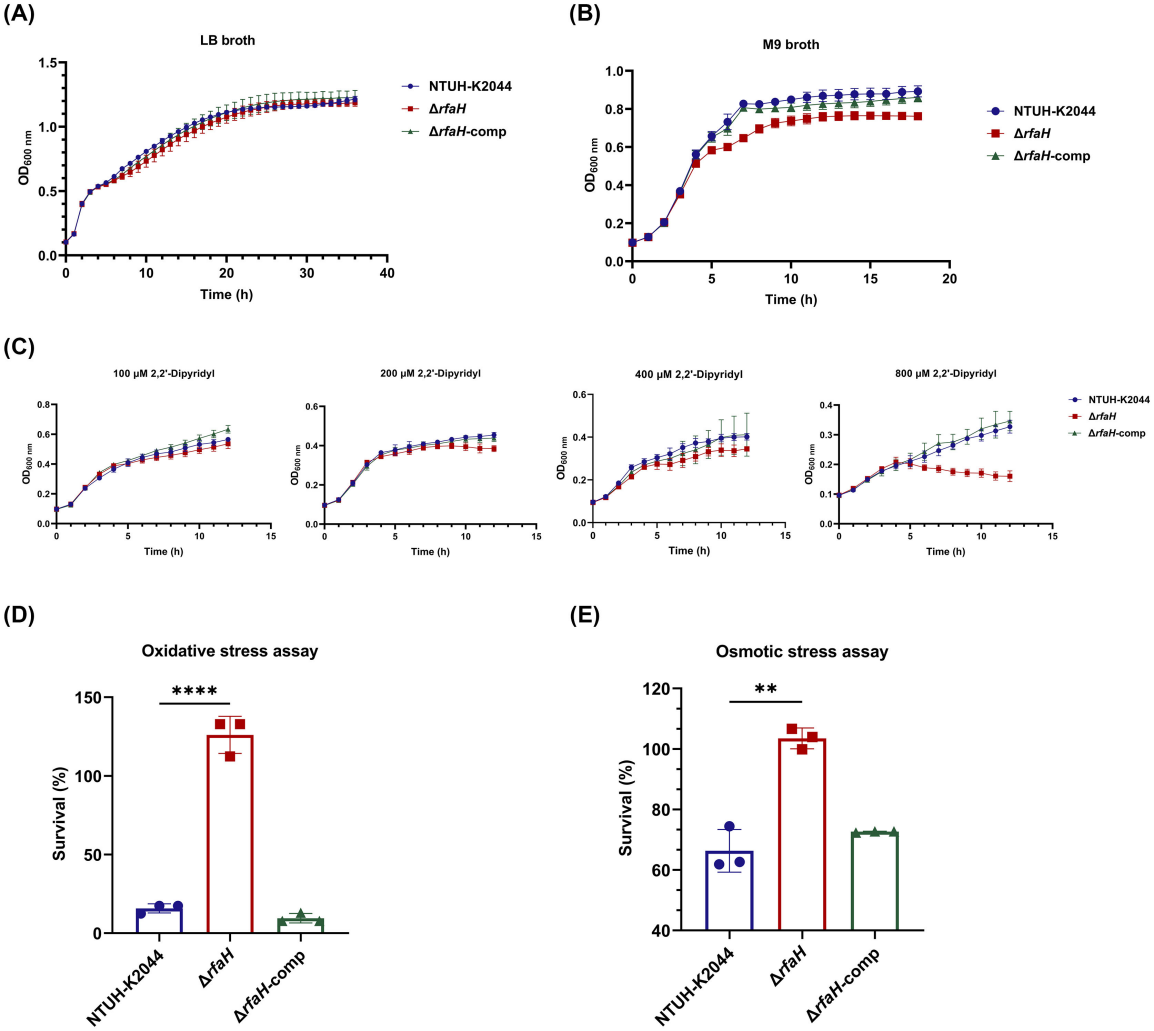
Growth ability and stress resistance assays. **(A)** Growth curves of NTUH-K2044, Δ*rfaH*, and Δ*rfaH*-comp in LB broth. The growth ability of Δ*rfaH* was comparable to the wild-type strain. **(B)** Growth curves of NTUH-K2044, Δ*rfaH*, and Δ*rfaH*-comp in M9 broth. Δ*rfaH* exhibited significantly slower growth since 4 h when compared to NTUH-K2044 (*P*<0.0001), and the maximum bacterial concentration in the stationary phase of Δ*rfaH* was significantly lower than that of NTUH-K2044 (*P*<0.0001). **(C)** Growth curves of NTUH-K2044, Δ*rfaH* and Δ*rfaH*-comp in LB broth in the presence of 100 μM, 200 μM, 400 μM or 800 μM 2,2’- dipyridine. Δ*rfaH* exhibited similar growth ability to NTUH-K2044 in the presence of 100 μM 2,2’-dipyridine, while a significant decreased growth of Δ*rfaH* was observed in the presence of 200 μM, 400 μM or 800 μM (Overall, *P*<0.05). **(D)** The oxidative stress assays. Δ*rfaH* showed increased resistance to 10 mM H_2_O_2_. **(E)** The osmotic stress assays. Δ*rfaH* showed increased resistance to 0.3 M potassium chloride. Data represent the mean of at least three independent experiments (in triplicate), and error bars represent the standard error of the mean. *P* values were calculated using student’s *t*-tests for statistical analyses, **P<0.01, ****P<0.0001.

Despite the obvious difference in colony morphology, Δ*rfaH* and NTUH-K2044 had similar growth ability in LB broth, a rich laboratory medium (**Figure 4A**). To investigate the involvement of *rfaH* in bacterial growth under nutrition-limited conditions, the growth kinetics of NTUH-K2044, Δ*rfaH*, and Δ*rfaH*-comp were compared. In the low-nutrition M9 broth, Δ*rfaH* exhibited significantly slower growth since 4 h when compared to NTUH-K2044 (*P*<0.0001), and the maximum bacterial concentration in the stationary phase of Δ*rfaH* was significantly lower than that of NTUH-K2044 (*P*<0.0001) (**Figure 4B**). In the iron-chelated LB broth, Δ*rfaH* and NTUH-K2044 exhibited similar stunted growth in the presence of 100 μM 2,2’-bipyridine (**Figure 4C**). While, as the concentration of 2,2’-bipyridine increased to 200, 400, or 800 μM, Δ*rfaH* displayed significantly decreased growth ability compared to the wild-type strain (overall, *P*<0.05, **Figure 4C**). Complementation with *rfaH* restored growth to a level resembling that of the wild-type parent. These results suggest that the deletion of *rfaH* leads to an impairment of overall growth kinetics under low-nutrient or iron-restricted conditions.

To assess the impact of *rfaH* deletion on bacterial responses to host stresses, NTUH-K2044, Δ*rfaH*, and Δ*rfaH*-comp underwent heat shock, acidic, osmotic, and oxidative challenges. As a result, Δ*rfaH* showed survival ability comparable to the wild-type strain after heat (Δ*rfaH* vs NTUH-K2044, *P*=0.9942 for 40°C, *P*=0.6154 for 50°C, and *P*=0.171 for 60°C, respectively) or acid (Δ*rfaH* vs NTUH-K2044, *P*=0.1141) treatments (**Supplementary Figures S2**, **S3**), indicating that the deletion of the *rfaH* does not affect bacterial response to heat-shock and acid adaptation. Unexpectedly, upon exposure to 10 mM H_2_O_2_ for 30_ min_, the survival rate of Δ*rfaH* was 126.1 ± 9.64%, notably higher than that of NTUH-K2044 (15.75 ± 2.35%, Δ*rfaH* vs NTUH-K2044, *P*<0.0001) and Δ*rfaH*-comp (9.52 ± 2.45%, Δ*rfaH* vs Δ*rfaH*-comp, *P*<0.0001) (**Figure 4D**). Similarly, Δ*rfaH* exhibited higher survival rates 103.5 ± 2.79% compared to the parental strain (66.33 ± 5.76%, Δ*rfaH* vs NTUH-K2044, *P*=0.0012) and Δ*rfaH*-comp (72.62 ± 0.17%, Δ*rfaH* vs Δ*rfaH*-comp, *P*<0.0001) in the presence of 0.3 M potassium chloride (**Figure 4E**). These results suggest that RfaH of *K. pneumoniae* is implicated in regulating bacterial growth in oxidative and osmotic conditions, while the underlying mechanism remains unknown.

### 3.5 RfaH plays an inhibitory role in biofilm formation

**Figure 5:**
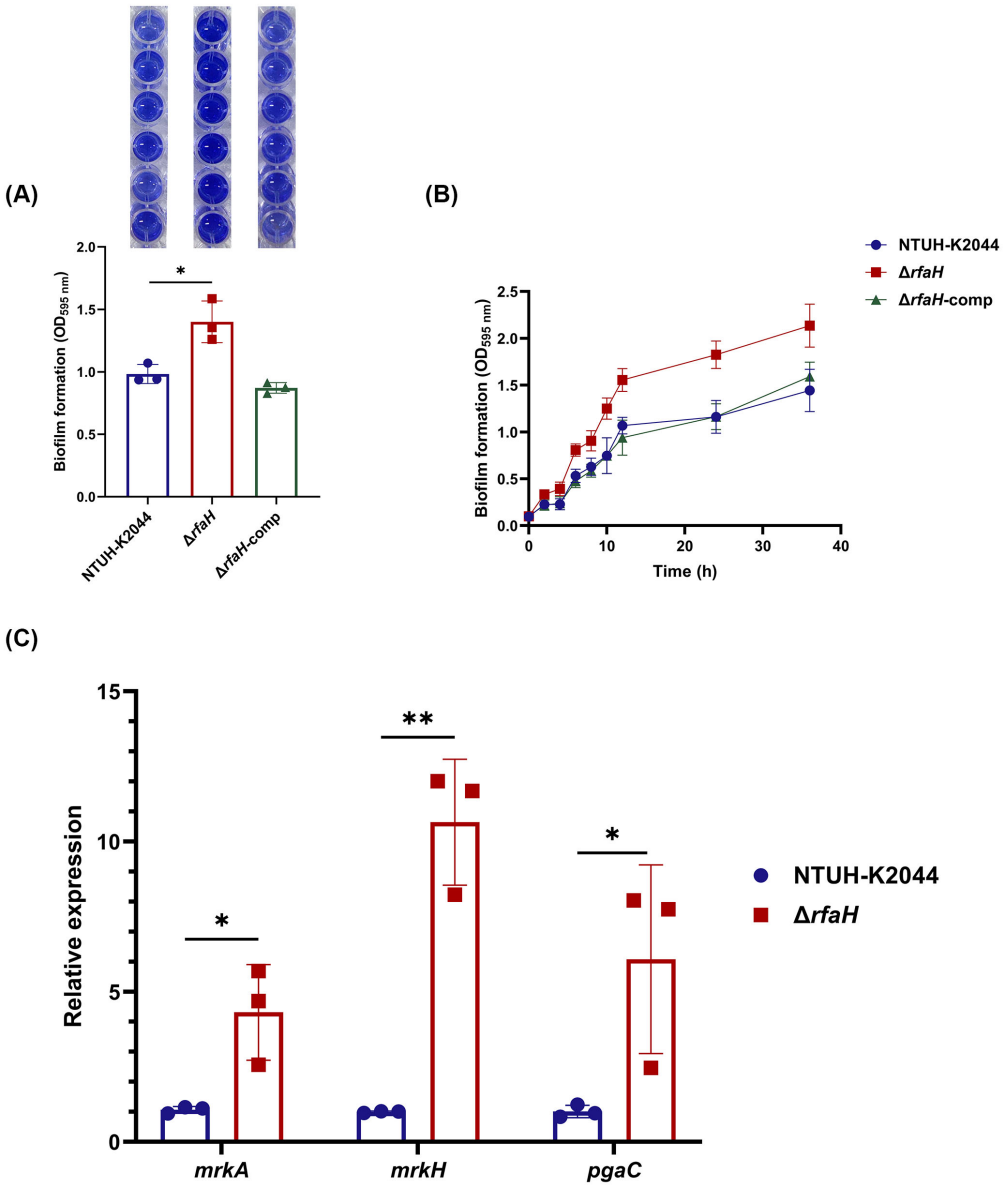
*rfaH* deletion improves biofilm formation. **(A)** Quantification of biofilm formation by crystal violet staining in 96-wells plate. This experiment was performed for three independent times, with six repetitions. A representative result is shown above the bar chart. **(B)** Continuous monitoring of the biofilm formation for 36 h. **(C)** The transcription level of genes associated with biofilm formation (*mrkA*, *mrkH*, and *pgaC*). Transcript abundance was measured using the 2^-ΔΔCt^ method with *16s rRNA* as a reference gene, and was normalized to the NTUH-K2044. Data represent the mean of at least three independent experiments (in triplicate), and error bars represent the standard error of the mean. *P* values were calculated using student’s *t*-tests for statistical analyses, *P < 0.05, **P<0.01.

Biofilm formation facilitates bacterial colonization and has been associated with reduced susceptibility to host immune responses (Zhu et al., 2021). To determine whether RfaH plays a role in the biofilm formation of *K. pneumoniae*, biofilm biomass formed by Δ*rfaH* and NTUH-K2044 was quantified using a crystal violet staining. Unexpectedly, the amount of biofilm in Δ*rfaH* displays a 29.8% increase compared to that of NTUH-K2044 (Δ*rfaH* vs NTUH-K2044,1.40 ± 0.137 vs 0.983 ± 0.062, *P*=0.0171, **Figure 5A**). Continuous monitoring from 0 to 36 hours further confirmed this result, with the biofilm production in Δ*rfaH* surpassing that of NTUH-K2044 from the 2nd hour and lasting till the 36th hour (*P*=0.0025, **Figure 5B**). The biofilm formation returned to the wild-type level in the complemented strain.

It was shown that type 3 fimbriae strongly promote biofilm formation in *K. pneumoniae* (Schroll et al., 2010). We found that levels of *mrkA* (encoding major pilus subunit MrkA) and *mrkH* (a transcriptional regulator of the *mrk* gene cluster) were 4.03-fold (*P*=0.0245) and 10.64-fold (*P*=0.0013) higher in Δ*rfaH* than in NTUH-K2044 (**Figure 5C**). In addition, analysis of the transcription level of *pgaC*, a gene encoding for Poly β-1,6-N-acetyl-D-glucosamine (PNAG) that is required for biofilm formation in *K. pneumoniae* (Chen et al., 2014), revealed a 6.08-fold (*P*=0.0492) increase in Δ*rfaH* (**Figure 5C**). These results suggest that RfaH may play an inhibitory role in the biofilm formation of *K. pneumoniae* by down-regulating the expression of some related factors, including type 3 fimbriae and PNAG production.

### 3.6 RfaH contributes to serum and phagocytosis resistance

**Figure 6:**
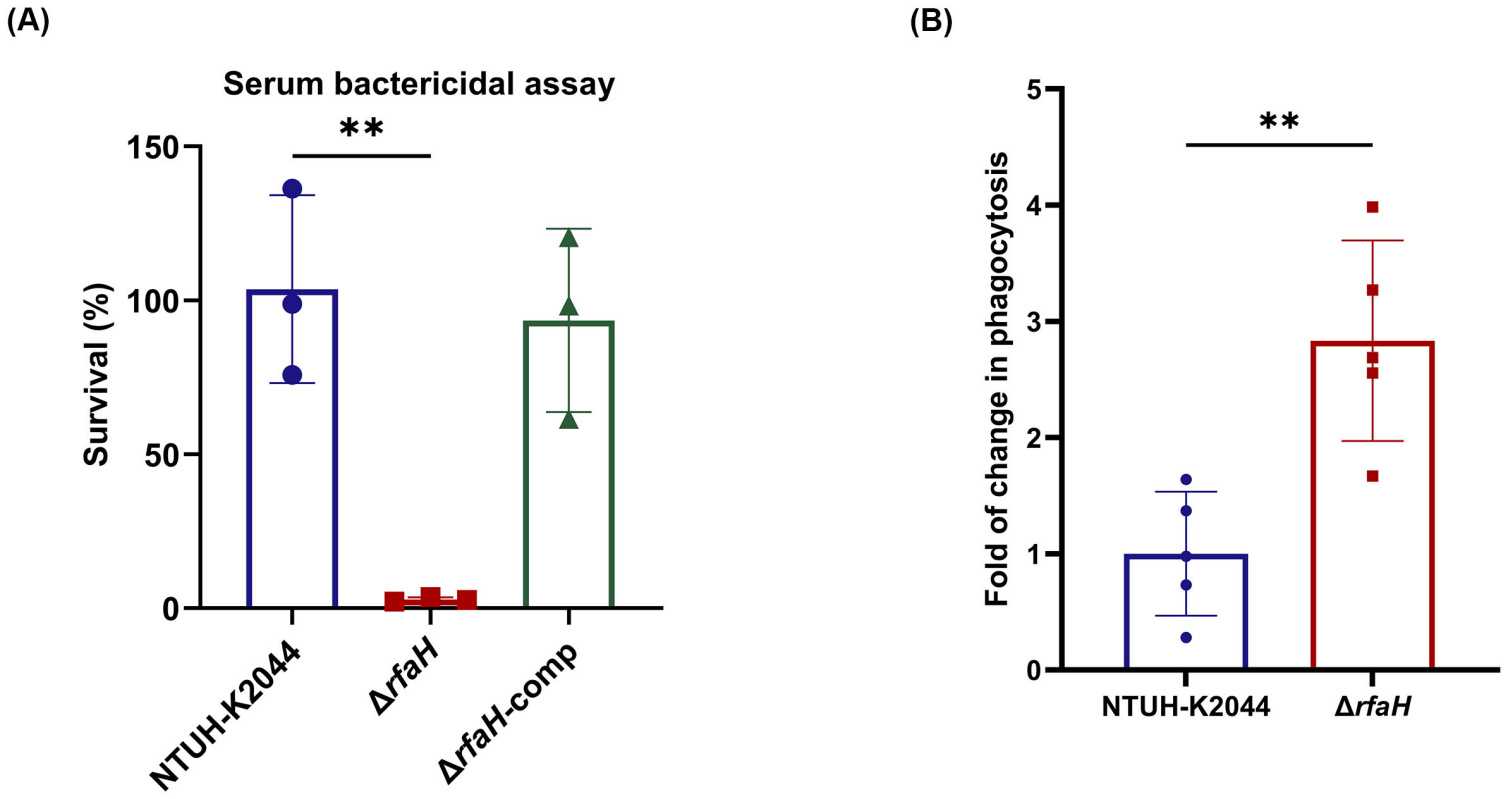
*rfaH* deletion reduces serum and phagocytosis resistance. **(A)** The serum bactericidal assays. Δ*rfaH* showed survival defects in 75% serum. Data represent the mean of at least three independent experiments (in triplicate), and error bars represent the standard error of the mean. **(B)** *in vivo* phagocytosis assays. Mice (five per group) were intranasally inoculated with 1 × 10^8^ CFU of NTUH-K2044 or Δ*rfaH*, respectively. A significantly increased engulfment of Δ*rfaH* by mouse macrophages was observed (*P*=0.0079). Each symbol represents 1 animal and error bars represent the standard error of the mean. *P* values were calculated using student’s *t*-tests for statistical analyses, **P<0.01.

To examine the involvement of RfaH in bacterial survival under serum exposure, serum bactericidal assays were performed. We found that the survival rate of Δ*rfaH* (2.82 ± 0.64%) was significantly lower than that of NTUH-K2044 (103.67 ± 24.9%, Δ*rfaH* vs NTUH-K2044, *P*=0.0046). The complementation of *rfaH* restored the survival rate to 106.42 ± 11.07% (Δ*rfaH* vs Δ*rfaH*-comp, *P*=0.0062). This result confirms the essential role of RfaH in resisting the complement-mediated killing of *K. pneumoniae* (**Figure 6A**). To assess directly the roles of RfaH in resisting early immune clearance *in vivo*, we performed alveolar lavage after the mice were infected with Δ*rfaH* or NTUH-K2044 by the intranasal route and counted the viable bacteria engulfed by alveolar macrophages. As a result, a significantly higher number of engulfed bacteria were obtained from mice infected by Δ*rfaH* (1.24 ± 0.59×10^5^ CFU/ml) when compared to those infected by NTUH-K2044 (3.51 ± 0.95×10^5^ CFU/ml, *P*=0.0079), yielding 1.67- to 3.99-fold more engulfment of Δ*rfaH* than the parental strain (**Figure 6B**), indicating that the absence of *rfaH* leads to significantly increased susceptibility to phagocytosis by macrophages. This result reveals a critical role of RfaH of *K. pneumoniae* in resisting phagocytosis in mice.

### 3.7 RfaH contributes to bacterial dissemination and colonization in the mouse pneumonia model

**Figure 7:**
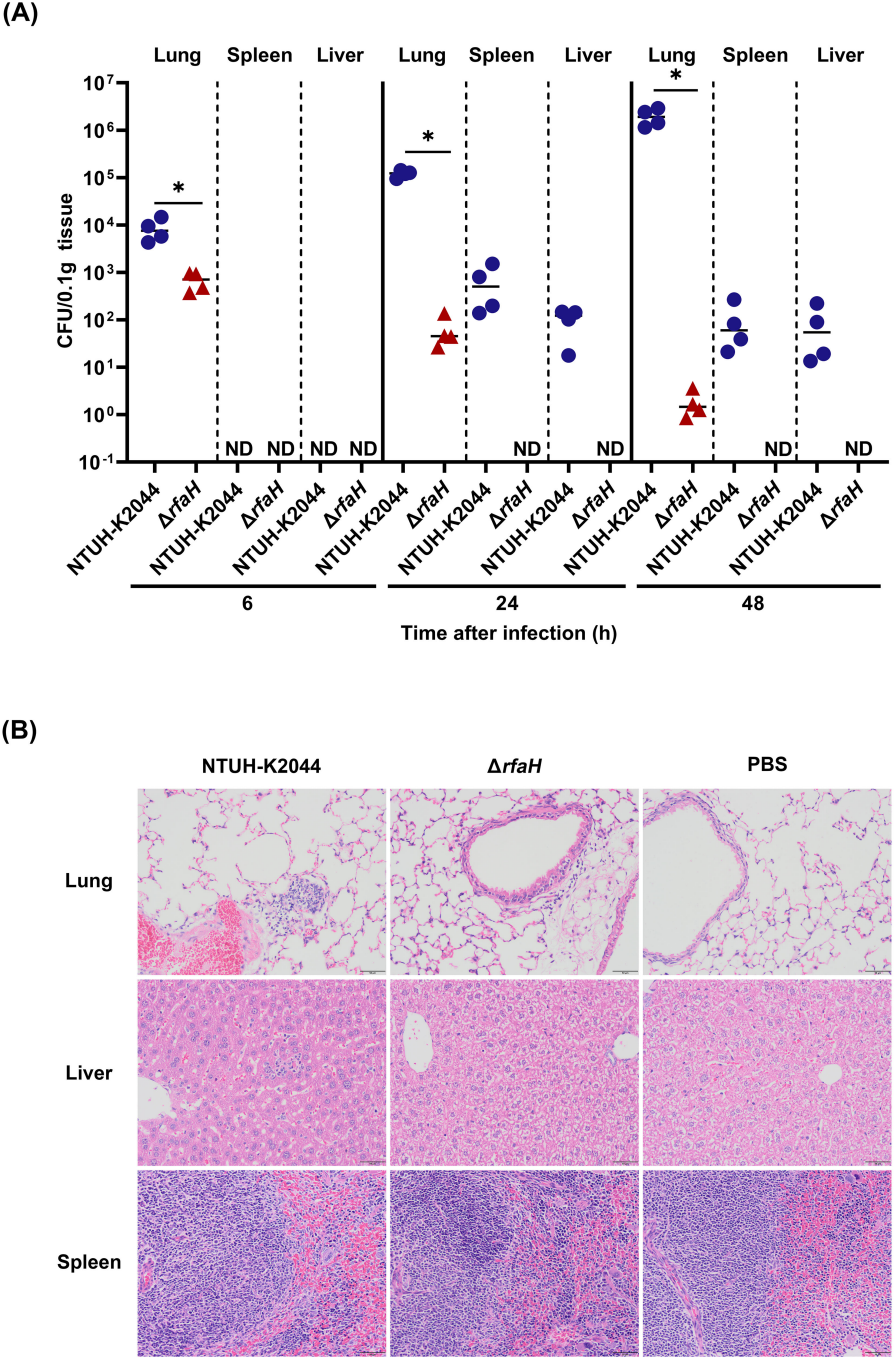
*rfaH* deletion decreases bacterial dissemination and colonization in mice by the intranasal route. Mice were intranasally inoculated with 1 × 10^5^ CFU of NTUH-K2044 or Δ*rfaH*, respectively. At the indicated times, mice were euthanized, and the lungs, spleens and livers were homogenized and plated for bacterial enumeration. **(A)** The organ burden assays after intranasal infection for 6 h, 24 h, and 48 h. Each symbol represents 1 animal, and short bars represent geometric means of each group. The Mann-Whitney test was used for statistical analyses, *P<0.05. ND, not detected. **(B)** Histopathology of lungs, spleens, and livers in mice after intranasal infection for 24 h.

To assess the role of RfaH in contributing to the pathogenesis of *K. pneumoniae*, BALB/c mice were intranasally inoculated with 1×10^5^ CFU of NTUH-K2044 or Δ*rfaH*, respectively. The bacterial loads in the lungs of mice infected by Δ*rfaH* were 1 log lower at 6 hours post-infection (hpi, NTUH-K2044 vs Δ*rfaH*, 8.6 (± 4.0) × 10^3^ vs 6.9 (± 2.6) × 10^2^ CFU/0.1g, *P*<0.05), 4 logs lower at 24 hpi (NTUH-K2044 vs Δ*rfaH*, 1.2 (± 0.2) × 10^5^ vs 6.4 (± 4.3) × 10^1^ CFU/0.1g, *P*<0.05), and 6 logs lower at 48 hpi than those in mice infected by NTUH-K2044 (NTUH-K2044 vs Δ*rfaH*, 2.0 (± 0.7) × 10^6^ vs 2.0 (± 1.0) × 10^0^ CFU/0.1g, P<0.05, **Figure 7A**). Δ*rfaH* was almost eliminated in the lung at 48 hpi. In spleens and livers, Δ*rfaH* was under the detection limit throughout the experiment. While the spleen titers in NTUH-K2044-infected mice were under detection limit at 6 hpi, but increased to 6.7 (± 5.6) × 10^2^ CFU/0.1g at 24 hpi, and 1.0 (± 1.0) × 10^2^ CFU/0.1g at 48 hpi (**Figure 7A**). Similar results were also observed in the livers, where the bacterial loads of NTUH-K2044 were undetected at 6 hpi, but increased to 1.0 (± 0.5) × 10^2^ CFU/0.1g at 24 hpi, and 8.6 (± 8.4) × 10^1^ CFU/0.1g at 48 hpi (**Figure 7A**). Altogether, these results showed that the absence of *rfaH* enhanced clearance of bacteria *in vivo*, indicating a critical role of RfaH in helping bacterial survival, dissemination, and maximal colonization in mice. Histopathology examination of the mice after 24 h of intranasal infection showed that NTUH-K2044 infection caused mild infiltration of inflammatory cells in the bronchi and adjacent tissues, along with dilated alveoli and congested blood vessels in the lung. In comparison, Δ*rfaH* infection caused scattered inflammatory cells in the alveolus, with no significant vascular congestion. In the spleen, the NTUH-K2044-infected mice exhibited dilated splenic blood sinusoids and an increase in the number of megakaryocytes, while Δ*rfaH*-infected mice showed slightly dilated splenic blood sinusoid with a minor megakaryocyte proliferation. Histopathology of the liver displayed no significant difference between two groups (**Figure 7B**). Considering the relatively short infection duration, the liver’s histopathological changes may not be evident, which is consistent with the bacterial load data.

### 3.8 RfaH contributes to bacterial colonization and pathogenicity in the intraperitoneal infection model

**Figure 8:**
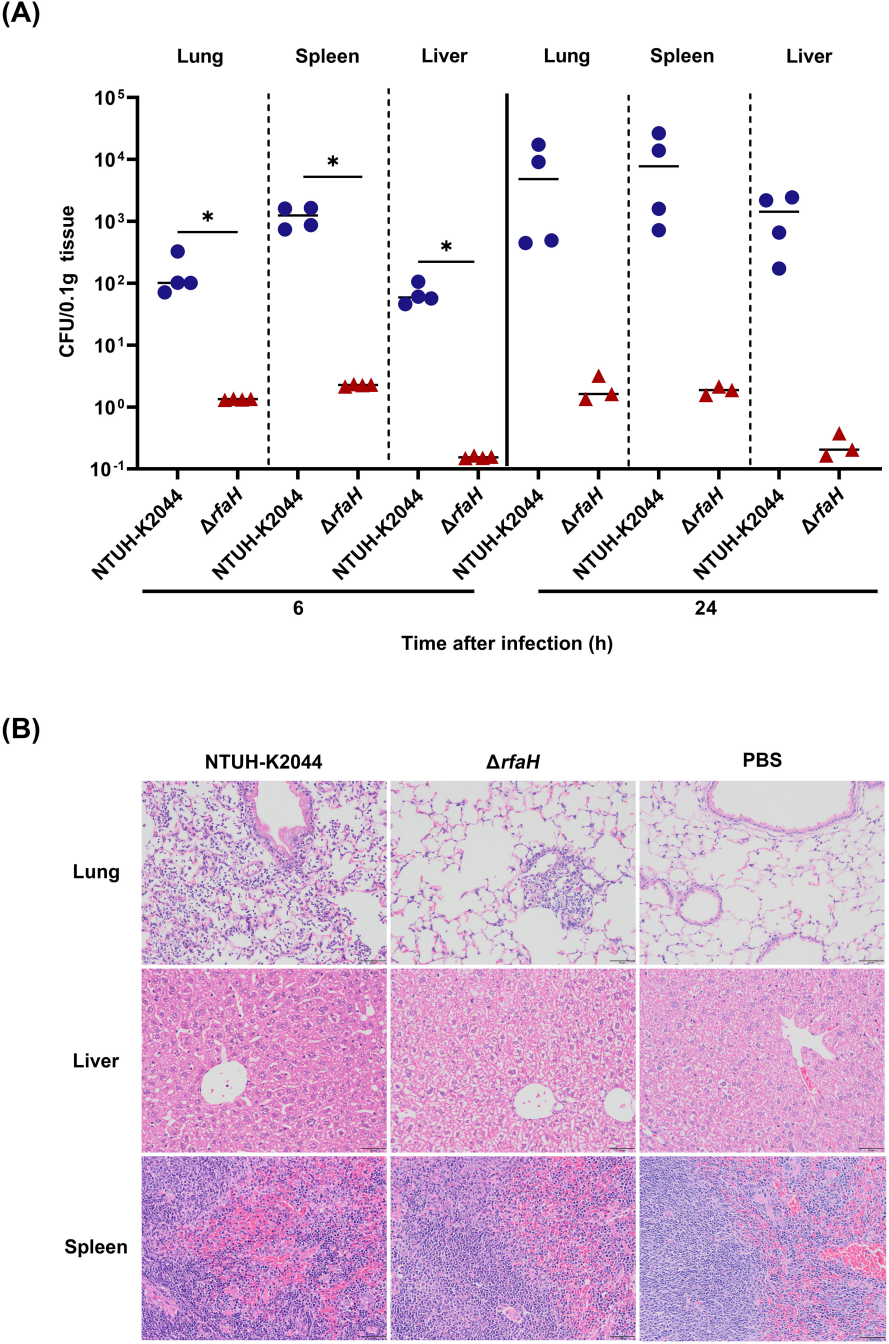
*rfaH* deletion decreases bacterial colonization and pathogenicity in mice by the intraperitoneal route. Mice were intraperitoneally inoculated with 1 × 10^4^ CFU of NTUH-K2044 and Δ*rfaH*, respectively. At the indicated times, mice were euthanized, and the lungs, spleens and livers were homogenized and plated for bacterial enumeration. **(A)** The organ burden assays after intraperitoneal infection for 6 h, 24 h, and 48 h. Each symbol represents 1 animal, and short bars represent geometric means of each group. The Mann-Whitney test was used for statistical analyses, *P<0.05. ND, not detected. **(B)** Histopathology of the lungs, spleens, and livers in mice after intraperitoneal infection for 24 h.

To further decipher the basis for the attenuation of mutant Δ*rfaH*, we analyzed bacterial colonization and pathogenicity by intraperitoneally inoculated BALB/c mice with 1 × 10^4^ CFU of NTUH-K2044 or Δ*rfaH*. Mice infected with Δ*rfaH* bore significantly (*P*<0.05) lower bacterial burdens in the lungs (NTUH-K2044 vs Δ*rfaH*, 1.5 (± 1.0) × 10^2^ vs 1.3 (± 0.1) × 10^0^ CFU/0.1g), liver (NTUH-K2044 vs Δ*rfaH*, 6.8 (± 2.3) × 10^1^ vs 1.6 (± 0.1) × 10^-1^ CFU/0.1g), and spleens (NTUH-K2044 vs Δ*rfaH*, 1.2 (± 0.4)× 10^3^ vs 2.3 (± 0.1) × 10^0^ CFU/0.1g) at 6 hpi. Similarly, after intraperitoneal infection for 24 h, the lungs, livers and spleens of mice infected with Δ*rfaH* also harbored decreased bacterial loads relative to those infected with NTUH-K2044 (**Figure 8A**). During this period, an increased number of NTUH-K2044 was observed in the lungs, spleens, and livers at 6 and 24 hpi, indicating bacterial multiplication of NTUH-K2044 through utilization of limited nutrients in mice, while Δ*rfaH* was rarely detected as a result of elimination by the immune system. All mice infected with NTUH-K2044 died during 24 to 48 h, so we were unable to compare the differences in bacterial loads between NTUH-K2044- and Δ*rfaH*- infected mice at 48 hpi. These results collectively suggest that RfaH contributes to bacterial fitness and colonization of *K. pneumoniae* in mice.

Histopathology examination showed that NTUH-K2044 infection caused moderate infiltration of inflammatory cells in bronchi and adjacent tissues, along with dilated alveoli and sporadic signs of hemorrhage in the lung of mice. In contrast, mice infected by Δ*rfaH* showed mild infiltration of inflammatory cells in the alveolus, with no significant sign of hemorrhage (**Figure 8B**). In the spleen, mice from both groups displayed dilated splenic blood sinusoids, accompanied by an increase in the number of megakaryocytes. There were no significant histopathological changes in the liver of both NTUH-K2044- and Δ*rfaH*-infected mice. The histopathology results are consistent with survival data and mouse pneumonia model data, which collectively highlights a requirement of RfaH for the full virulence of *K. pneumoniae* in mice.

## 4 Discussion

RfaH is well studied as an important pathogen virulence factor in *E. coli* by promoting LPS synthesis, K15 capsule and α-hemolysin (Nagy et al., 2002) and *S. enterica* by impacting the production of LPS (Nagy et al., 2006). In *Y*. *pestis*, RfaH contributes to the expression of LPS but has little effect on bacterial acute virulence (Hoffman et al., 2017). Previous studies suggested an essential role of RfaH in the CPS production in *K. pneumoniae*, and its involvement in bacterial fitness in mice (Bachman et al., 2015; Mike et al., 2021). In our work, we demonstrate that RfaH plays a critical role in the CPS synthesis of *K. pneumoniae* NTUH-K2044, a hvKp characterized by hyper-capsule. The loss of RfaH results in reduced bacterial survival, dissemination, colonization, and full virulence in mice, which is likely an indirect consequence of CPS deficiency. Collectively, this makes RfaH a promising drug target, especially for treating infections by hvKp.

In this study, we confirmed that RfaH contributes to CPS biosynthesis by directly promoting the transcription of *cps* operon in *K. pneumoniae*, as found in *E. coli* (Bailey et al., 1997). It is known that RcsAB directly activates the transcription of *cps* operon by binding to a DNA sequence called the RcsAB box located upstream of the *cps* gene cluster in several bacterial organisms (Walker and Miller, 2020). We found here that the significantly reduced expression of *cps* operon as a result of *rfaH* deletion did not bring changes to the expression of *rcsA*. This finding indicates that RcsAB and RfaH regulate the transcription of *cps* operon independently, and the decreased expression of *cps* operon did not lead to upregulation of *rcsA* as a response in our case.

A previous study revealed that the transcription of *rfaH* in *Salmonella enterica serovar Typhi* shows a growth-phase dependent regulation, with maximal expression at the late exponential and stationary phase (Rojas et al., 2001) and that nitrogen limitation increases the transcription of the *rfaH* (Bittner et al., 2002). That is, RfaH may be involved in regulating the utilization of nitrogen sources, particularly during the nutrient deficiency phase. In this study, the growth rate of Δ*rfaH* significantly decreased when compared to its parental strain during the logarithmic and stationary phases in the M9 medium. We reasoned that the absence of RfaH may lead to dysregulation or suboptimal utilization of nitrogen sources in M9 medium, causing bacterial growth retardation. The specific mechanism of RfaH in regulating energy metabolism remains to be further studied. In the iron-limited condition, the lower growth kinetics of Δ*rfaH* was most likely result from its CPS deficiency, due to the close correlation between the CPS biosynthesis and environmental iron availability in hvKp (Thomas A. Russo, 2019).

In stress-response assays, Δ*rfaH* showing higher survival rates under oxidative and osmotic stress conditions were unexpected. In response to oxidative stress, bacteria can deploy the OxyR and SoxRS systems to transcriptionally activate genes either to protect or repair damage caused by intracellular ROS accumulation in *K. pneumoniae* (Anes et al., 2021). In our work, the transcription levels of *soxS* and *oxyR* were unchanged after the deletion of *rfaH* (Data not shown), suggesting that *rfaH* may respond to oxidative stress in a *soxS-* or *oxyR-*independent way. It has been shown that outer membrane porin OmpK36 contributes to high osmotic resistance by regulating membrane permeability (Wang et al., 2022). While the absence of *rfaH* did not change the expression of *ompK-36* under high osmotic stress in this work (Data not shown). We speculate that the defects in LPS caused by *rfaH* deletion might disturb the assembly of outer membrane proteins, resulting in a decrease in membrane permeability (Tian et al., 2022), which may become a growth advantage in oxidative or osmotic stress conditions.

Biofilm formation is a critical factor in the pathogenesis of *K. pneumoniae*, influenced by various aspects such as environmental conditions, flagella, fimbriae, capsule, and quorum sensing systems (Clegg and Murphy, 2016; Wang et al., 2020). Our study found that Δ*rfaH* significantly increased biofilm formation, which would be most likely caused by unmasking of proteinaceous adhesins as a result of CPS loss (Sahly et al., 2000). Type 3 fimbriae encoded by the *mrk* operon (*mrkABCDFJ*) is known to strongly promote biofilm formation in *K. pneumoniae* (Schroll et al., 2010). *mrkH* acts as a regulatory factor facilitating the transcription of the *mrk* operon (Johnson et al., 2011). The simultaneous increased expression of *mrkA* and *mrkH* in Δ*rfaH* indicates that RfaH is likely to participate in the repression of the *mrk* operon in a *mrkH*-dependent way. PNAG encoded by the *pgaABCD* operon is a bacterial surface component required for biofilm formation (Wang et al., 2005). The deletion of *pgaC* results in a significantly reduction in the biofilm formation in *K. pneumoniae* (Chen et al., 2014; Huang et al., 2014). In our study, we observed an up-regulated expression of *pgaC* after the deletion of *rfaH* (data not shown), suggesting an inhibitory role of RfaH in the expression of *pgaC*. The upregulated expression of type 3 fimbriae and PNAG gives alternative explanations for the increased biofilm formation in Δ*rfaH*. As a transcriptional elongation factor, RfaH displays inhibitory functions in the *mrk* and *pga* operons is unexpected. Efforts are underway to further investigate the underlying mechanisms.

Complement proteins are important components of host innate immunity, and both CPS and LPS play crucial roles in bacterial resistance to complement-mediated killing in human serum (Short et al., 2020). The abolished CPS production and truncated LPS in Δ*rfaH* could explain its impaired survival capabilities in human serum. Meanwhile, Δ*rfaH* showed significant susceptibility to macrophage phagocytosis during lung infection in mice. It was reported that HMV blocks adherence and internalization by macrophages (Mike et al., 2021). Therefore, the decreased HMV and the increased complement-mediated opsonization as a result of diminished CPS in Δ*rfaH* may jointly lead to an enhanced macrophage phagocytosis *in vivo*. These results echo the requirement of RfaH in serum resistance and demonstrate its essential role in bacterial defense against phagocytosis by macrophages. The organ burden assays indicated a higher bacterial clearance rate of Δ*rfaH* in the lungs in the intranasal infection model, and that Δ*rfaH* almost could not spread to the livers and spleens of infected mice. Similarly, Δ*rfaH* was quickly cleared by livers, spleens, and lungs in the intraperitoneal infection model. The decreased bacterial colonization, dissemination and pathogenicity explained the attenuation in virulence of Δ*rfaH* in mice. These results collectively revealed that RfaH helps bacterial survival and maximal colonization in the host, and ultimately contribute to the full virulence of hvKp. In conclusion, our work demonstrates that RfaH positively regulates the pathogenicity of hvKp, by promoting the CPS production to mediate successful colonization and full virulence. Therefore, developing small molecule inhibitors targeting RfaH could have great potential to treat *K. pneumoniae* infections. This is of particular significance for hvKp, as increased capsule production is an established hvKp-specific virulence factor.

## Data Availability

The raw data supporting the conclusions of this article will be made available by the authors, without undue reservation.
