# Supplementary material for: RfaH contributes to maximal colonization and full virulence of hypervirulent Klebsiella pneumoniae
Source: Front Cell Infect Microbiol. 2024 Sep 17;14:1454373. doi: 10.3389/fcimb.2024.1454373 (PMC11448354; doi:10.3389/fcimb.2024.1454373)
Supplement: Supplementary file 1 [file Table1.docx]

Supplementary Material

RfaH contributes to maximal colonization and full virulence of hypervirulent *Klebsiella pneumoniae*

Yichuan Qiu^1^, Li Xiang^2^, Ming Yin^2^, Chengju Fang^2^, Xiaoyi Dai^2^, Luhua Zhang^2,*^, Ying Li^2,*^

1. Department of Clinical Laboratory, Hospital of Chengdu Office of People’s Government of Tibetan Autonomous Region, Chengdu, Sichuan, China

2. The School of Basic Medical Sciences, Southwest Medical University, Luzhou, Sichuan, China

*Correspondence: Ying Li, Lying1019@swmu.edu.cn or Luhua Zhang, [zhluhua@swmu.edu.cn](mailto:zhluhua@swmu.edu.cn).

**Table S1 Primers used in this study.**

| **Primers** | **Sequence (5’ - 3’)** | **Application** |
| --- | --- | --- |
| *rfaH-*L1 | CGACTCTAGAGGATCGCGGCCGCGCACCACTCCTGGAAATCCAG | Amplification of the upstream flanking region of *rfaH* |
| *rfaH-*L2 | GGAAGTCGGTATTTTTAACGCTCCGTTTGCAATACAGTAAGTACC |  |
| *rfaH-*R1 | GGTACTTACTGTATTGCAAACGGAGCGTTAAAAATACCGACTTCC | Amplification of the downstream flanking region of *rfaH* |
| *rfaH-*R2 | CGGTACCCGGGGATCGCGGCCGCGGCGTTAATCTGACCAGTTCGC |  |
| *rfaH-*L1long | GCCAAGAATGGTTGCCGGATCG | Detection of Δ*rfaH* |
| *rfaH-*R2long | AGAGCACAGAGAAGAGTAATACC |  |
| *pgpA-*L | CGACTCTAGAGGATCGCGGCCGCCGGCGCACGTATCGATCTCG | Amplification of the upstream flanking region of *rfaH* |
| *pgpA-*R | GGCGTGGTTTCCCCGGCAGCGAGCTCGATAGGGCCCTATGTAGCGTTGCCGGGGAGAGG |  |
| *yajO-*L | CGGTACCCGGGGATCGCGGCCGCGCAGCGAAGAGATCGTTGGC | Amplification of the downstream flanking region of *rfaH* |
| *yajO-*R | CCTCTCCCCGGCAACGCTACATAGGGCCCTATCGAGCTCGCTGCCGGGGAAACCACGCC |  |
| *pgpA-*Llong | ACCTGGCGACATCGGCTATCG | Detection of Δ*rfaH*-comp |
| *yajO-*Llong | TATCCAGCACGCCATTGAAGG |  |
| *rfaH-*Lc | CGGCAACGCTACATAGGGCCCTCAGAAAGTCGCGAAGATCGT | *rfaH* cloning |
| *rfaH-*Rc | GCAGCGAGCTCGATAGGGCCCAACGTCTGCTGCTGGAGACCG |  |
| *kan*-L | CACCGAGGCAGTTCCATA | Detection of pKO3-Km |
| *kan*-R | CTCTTCCGACCATCAAGC |  |

**Table S2 Primers used for qRT-PCR in this study.**

| **Primers** | **Sequence (5’ - 3’)** | **Target gene** |
| --- | --- | --- |
| 16S rRNA-F | TGATCATGGCTCAGATTGAACG |  |
| 16S rRNA-R | GCAGTTTCCCAGACATTACTCAC |  |
| *wzi-F* | CCAGTCGTTAGAATTAGGTGCGT | *wzi* |
| *wzi-R* | CTCTGTCCCGGTATTATCTTTGC |  |
| *manC-F* | GACTTCATCAGCATCCCGCA | *manC* |
| *manC-R* | CATCAAGACCGACCACCACC |  |
| *rcsA*-F | ATCCATTTTGACCGCTATTTGC | *rcsA* |
| *rcsA*-R | CTGCCCTACACTGGTGTTTTTG |  |
| *wzt*-F | GCTGAAGGGGCGGAAATAT | *wzt* |
| *wzt*-R | GGCAACCAGGCCAAGAGAG |  |
| *wbbY*-F | CAAGCGATTCACTTCACTCTCA | *wbbY* |
| *wbbY*-R | TAACTACCGCAGTTTCCGTCCT |  |
| *glf*-F | ACTTTTGTCGGTCGTCTTGGAA | *glf* |
| *glf*-R | AACACAGGCATTGGCTGATTTT |  |
| *waaA*-F | GGCTCCTGCCTATCGCAAAC | *waaA* |
| *waaA*-R | GAATGGCGGCTAAGGTCTCG |  |
| *waaF*-F | ATCGGCATTAGTCCCTTTCTTTG | *waaF* |
| *waaF*-R | GGCGTCTTTGTCCAGCACCC |  |
| *mrkA*-F | CCCTGACTGAAGTTAAAGCGGCA | *mrkA* |
| *mrkA*-R | CCCAGTTTGCTTACGTCATCCTG |  |
| *mrkH*-F | TTCATATTCAGACCCATCGCAA | *mrkH* |
| *mrkH*-R | CCGCATTAAACTCTATTTTCCC |  |
| *pgaC*-F | TCCGCCACTGGGACATCTTT | *pgaC* |
| *pgaC*-R | CGCTGCTTCCATAACCCTTTGA |  |


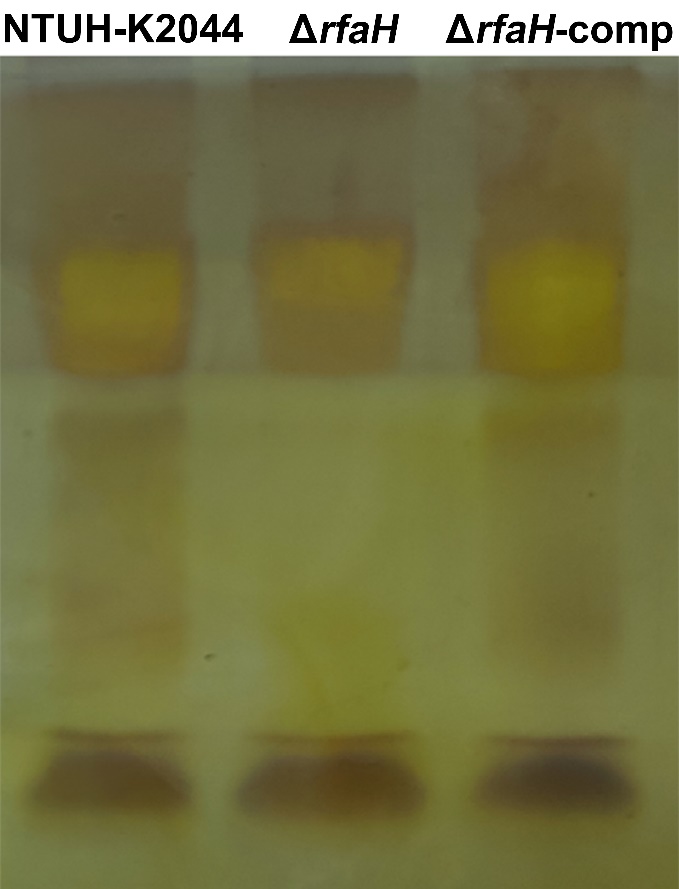


**Figure S1** SDS-PAGE and silver staining of LPS. A lower level of LPS in Δ*rfaH* was observed when compared to NTUH-K2044 and Δ*rfaH*-comp.


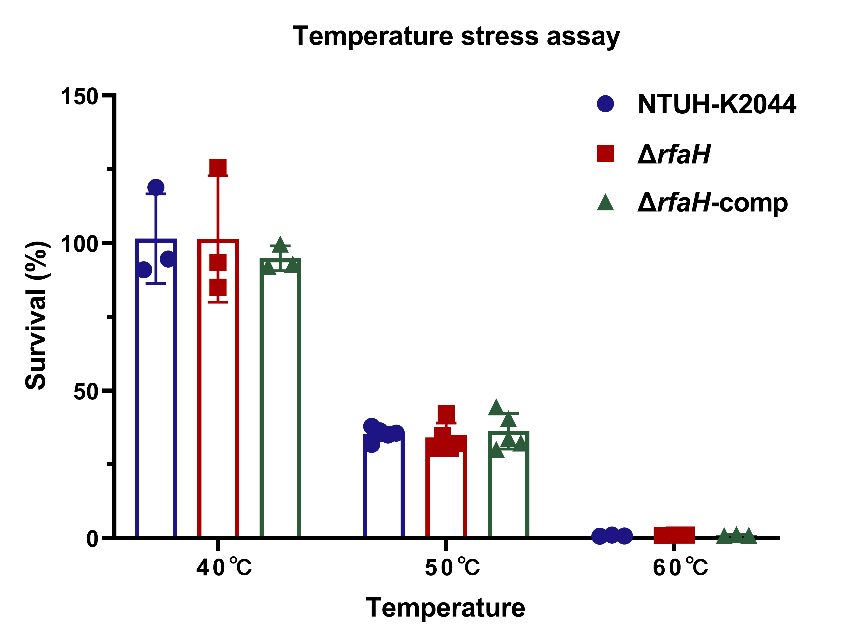


**Figure S2** *rfaH* deletion does not affect bacterial heat resistance. The temperature stress assay was conducted at 40℃, 50℃, and 60℃, respectively. Data represent the mean of at least three independent experiments (in triplicate), and error bars represent the standard error of the mean. *P* values were calculated using student's *t*-tests for statistical analyses.


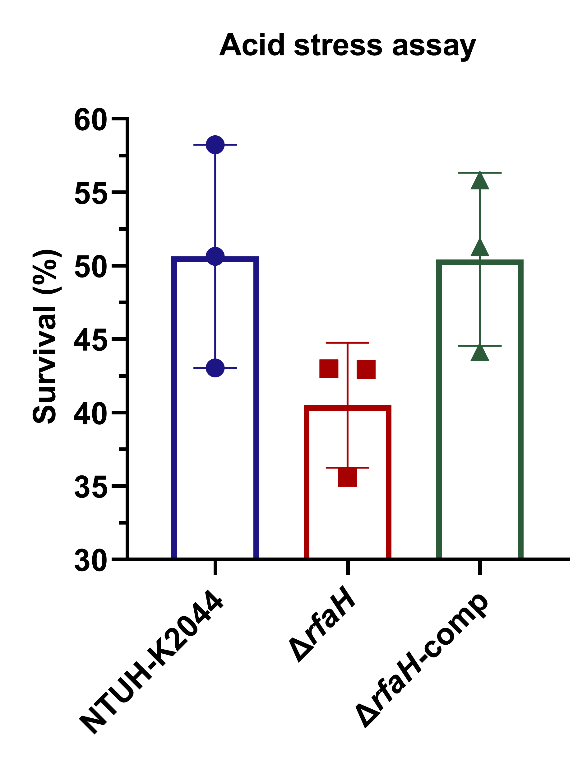


**Figure S3** *rfaH* deletion does not affect bacterial acid resistance. Data represent the mean of at least three independent experiments (in triplicate), and error bars represent the standard error of the mean. *P* values were calculated using student's *t*-tests for statistical analyses.
